# General Overview of Psychotherapeutic Practice in Poland. Results from a Nationwide Survey

**DOI:** 10.1007/s11126-016-9480-9

**Published:** 2016-11-02

**Authors:** Hubert Suszek, Lidia Grzesiuk, Rafał Styła, Krzysztof Krawczyk

**Affiliations:** Faculty of Psychology, University of Warsaw, Ul. Stawki 5/7, 00-183 Warsaw, Poland

**Keywords:** Psychotherapists, Psychotherapy, Poland, Professional practice, Theoretical orientation, Nationwide survey

## Abstract

A total of 1196 persons conducting psychotherapy in Poland fully completed a nationwide online survey (or, alternatively, a paper and pencil enquiry) concerning their education, training, experience, and clinical work (professional environment, patients treated). The results are described in detail and compared with findings of similar studies from other countries. Among the primary findings were: (1) psychotherapy in Poland is conducted mostly by women (80 %); (2) almost all participants have an MA degree (91 %), including 75.2 % having graduated in psychology; (3) the therapists are well trained (mean number of training hours is above 942) and established (average experience is about 9.8 years), however, more than half of the therapists have no type of certificate; (4) 54 % of respondents identify with the integrative or eclectic orientation and, simultaneously, for 48.6 % of the therapists the most important approach is either psychodynamic or psychoanalytic; (5) the most common form of therapy is individual psychotherapy in private practice; (6) the majority of the therapists treat adult patients with anxiety or personality disorders. In sum, the results show that psychotherapeutic practice is well established in Poland and many indices are similar to those found in Western countries.

In the last few years we have observed significant and rapid development of psychotherapy in Poland, especially in the private practice sector. This kind of practice was practically non-existent twenty years ago. During the communist regime in Poland, there were limited possibilities for travelling to Western countries and for acquiring expertise in psychotherapy, in gaining training or in inviting lecturers and trainers to workshops. However, in those difficult times some prominent psychiatrists and psychologists elaborated and organized several psychotherapy training programs for major psychotherapy approaches (e.g. psychoanalysis, humanistic therapy, systemic family therapy and community psychiatry, see [1–3]. The political transformation in 1989 of Poland into a democracy and free market allowed psychotherapy to arise. Poland’s entrance into the European Union in 2004 resulted in new possibilities.

Today, the value of psychotherapy is being continuously acknowledged. It seems that the profession of the psychotherapist has also gained significant popularity. In the last few years many training programs have developed that are connected with a variety of psychotherapeutic approaches whose members belong to many psychotherapeutic associations (see [4]). In 1991 and 1994, the two largest and earliest associations, i.e. the Polish Psychiatric Association and the Polish Psychological Association, designed the criteria and requirements for certifying psychotherapists and supervisors. Soon other psychotherapeutic associations developed their own regulations. Some of the associations started collaboration with international psychotherapeutic institutions and currently provide training at a level at which internationally recognized certificates are issued. Since 2005 the Polish Ministry of Health has been working on an act for other medical professions which also encompasses the profession of the psychotherapist.

Today, there are about 25–30 long-term postgraduate training programs in Poland which last at least 4 years and offer certification as a psychotherapist. These programs usually require about 1200 h of theoretical and practical classes, including 100–250 h of training in psychotherapy, 1–2 years of clinical practice, and 150–200 h of supervision [4]. Moreover, a countless number of short-time training and courses is available. There are currently over 80 psychotherapeutic associations in Poland.

It is difficult to precisely define the total number of psychotherapists in Poland. Psychotherapy is typically practiced by members of several different professions (psychologists, psychiatrists, educators, etc.) and there is no single organization that includes all of these professionals. According to data gathered by Suszek et al. [5], there are approximately 1500 psychotherapists certified by 10 major psychotherapeutic associations in Poland, but the total number of people conducting psychotherapy remains unknown. Despite the significant development of the profession, there is still no law specifying the competences that are needed for successfully completing psychotherapy training and practicing psychotherapy in Poland. It is now legal in Poland to set up a psychotherapy practice without having any professional competences (only administrative and fiscal registration is necessary). The only domain for which there are regulations is medical care, as the national health insurance institution pays only for psychotherapy that is conducted by psychologists or psychiatrists who are certified therapists or candidates in selected psychotherapeutic associations. Due to insufficient regulations in the psychotherapeutic profession and training, there is relative freedom for practicing psychotherapy in Poland. There is also a lack of knowledge as to what this psychotherapy practice looks like in Poland and who the therapists are. The lack of legal regulations for psychotherapy is also connected with the risk of non-scientific, inappropriate and harmful practice.

There is very little research data which shows how psychotherapy used to be practiced in the past in Poland and how it is practiced now. We identified only 6 studies which directly addressed this problem. These studies are characterized by low sample sizes, thus making it almost impossible to generalize their results and to capture the changes that have taken place in the development of psychotherapy in Poland: Kuliszkiewicz, Dominik, Madej, Rogiewicz, Sęk & Szydlik – N unknown [6]; Łapiński, Malatyńska, Orwid, Osuchowska & Piotrowski – *N* = 57 [7], Fichter & Wichten – *N* = 5 [8], Czabała & Mroziak – N unknown [9]; Czabała & Brykczyńska – *N* = 34 [10].

The most recent study was conducted in 2001 by Czabała and Brykczyńska [10] among 24 Polish psychotherapists. The study was a part of a larger project titled “Psychotherapy in Europe” and was run on 181 psychotherapists employed in public health service from 7 countries, including Poland. The researchers found that the vast majority of Polish psychotherapists in the study had an academic degree and postgraduate training in psychotherapy, and that almost all of them were psychiatrists or psychologists. There were slightly more psychiatrists than psychologists working as psychotherapists. In comparison with other countries, Polish psychotherapists had a shorter training period (2.8 years on average as compared with 5.1 years on average in other countries) and less supervision (3.1 vs 6.5 years as compared to other countries). Psychodynamic and cognitive-behavioral frameworks were the modalities which the therapists used most frequently. The majority of respondents (96 %) declared that they changed theories depending on their patients’ needs.

Due to the outdated status of previous research on psychotherapy in Poland, the small sample sizes and observations of significant developments of the discipline, it is important to explore the current status of psychotherapy practice. We wanted to provide a detailed description of the characteristics and practices of therapists in Poland. Specifically, we wanted to provide answers as to (a) how Polish therapists were trained (education, training programs, training therapy, supervision, certificates), and (b) how they practice (approaches, forms of treatment, work setting, patients). We believe that our study can be of interest to many clinicians around the world because it is the first nationwide empirical study presenting the field of psychotherapy in the sixth most populated country in the European Union. To our knowledge this is also the first published research study on the psychotherapy profession conducted in Eastern Europe.

## Method

### Research Instrument

For the purpose of the study we constructed a self-administered survey consisting of 40 items covering various aspects of therapists’ professional characteristics, educational background, work experience, work setting, work roles, case load, training and specialization, theoretical orientation, supervision and questions about demographic variables. Most of the items were designed as checklists. The mean time of completion of the survey was 14.2 min (SD = 7.7). The survey was conducted in two versions: a paper version and in the form of an online survey.

### Procedure and Participants

Various methods of data collection were used. First, we put up ads and invitations on the websites of the largest associations connected with psychotherapy (e.g. Polish Psychological Association, Polish Psychiatric Association).

Second, we conducted an extensive Internet search for e-mail addresses to various associations and psychotherapy training centers (searching for phrases such as “psychotherapy center”). We also searched for institutions for which people offering psychotherapy might work, e.g. public psychotherapy institutions, psychiatric hospitals and clinical psychology or psychiatry departments at universities. In sum, we collected 1500 such addresses. We sent email invitations to those email addresses, asking for them to be forwarded to workers or members known to be engaged in therapy, counseling, or cognate practices. The invitations directed the respondents to a secure website where they could complete the survey.

Third, we assumed that the majority of persons who offer psychotherapy in private practice use some kind of promotion of their services in the Internet. Through a similar Internet search we collected another 1500 of such addresses (with words such as *psychotherapist; psychotherapy practice*) and we sent email invitations to those email addresses. We also used the snowball technique by asking respondents to forward the Internet address of the survey to their colleagues. Additionally, several dozen questionnaires were distributed during conferences dedicated to psychotherapy and were drawn from the networks of professional colleagues. The online and paper survey contained an introductory letter which explained the purpose of the study and guaranteed anonymity of the responses.

Fourth, we put up ads and invitations on the webpages of popular social networking pages (e.g. *Facebook, Goldenline*).

Between January and December 2012, 1838 participants registered and at least partially completed the survey. The response rate is difficult to estimate due to various institutional addresses and ads on the Internet. The participation was voluntary, anonymous, and without remuneration.

Among the group of 1838 subjects, 642 participants (35 %) stopped filling out the questionnaire at different stages of that questionnaire. All of the further analyses presented in the Results section were conducted on the sample of 1196 persons that had filled out all of the required items.^1^

## Results

Among the participants, 79.9 % (*N* = 956) were women and 20.1 % (*N* = 240) were men. The therapists’ age varied from 26 to 93 years, with an average age of 40.2 years (SD = 9.6).

### Education, Training and Experience of the Therapists

The great majority of participants in this study had an MA degree (91 %). Three out of four surveyed therapists had graduated in psychology, and 13 % (*N* = 135) of the therapists had completed two or three faculties (see Table 1).

**Table 1:** Education of the therapists

Education	N	%
Degree		
MA	1087	91.0 %
PhD	84	7.0 %
Other	24	2.0 %
Missing data	1	0.1 %
Completed faculty^a^		
Psychology	876	75.2 %
Pedagogy	155	13.3 %
Medicine	63	5.4 %
Sociology	43	3.7 %
Philology	31	2.7 %
Resocialization	20	1.7 %
Philosophy	18	1.5 %
Theology	14	1.2 %
Law	6	0.5 %
Political studies	7	0.6 %
Management	5	0.4 %
Economics	5	0.4 %
Other	62	5.3 %
Missing data	31	2.6 %

Frequencies (%) do not include missing data

^a^More than one answer was allowed, that is why the sum does not total 100 %

The respondents’ experience ranged from less than 1 to more than 45 years of practice, with a mean experience level of 9.8 years (SD = 7.8). The respondents’ professional experience was classified into 6 categories of career level based on the criteria proposed by Orlinsky & Rønnestad [19]. The results of this classification are included in Table 2.

**Table 2:** Experience of the therapists

Experience of the therapists	N	%
Career level		
Novices (less than 1.5 years of experience)	14	1.2 %
Apprentice (1.5–3.5 years of experience)	171	14.6 %
Graduate (3.5–7 years of experience)	313	26.7 %
Established (7–15 years of experience)	456	38.8 %
Seasoned (15–25 years of experience)	146	12.4 %
Senior (25–45 years of experience)	74	6.3 %
Missing data	22	1.8 %
Personal therapy		
Yes	1046	87.5 %
No	150	12.5 %
Supervision		
Only group	408	34.1 %
Only individual	221	18.5 %
Both forms	517	43.2 %
None	50	4.2 %
Possesses (a) psychotherapy certificate(s)		
Yes	521	43.6 %
No	675	56.4 %

Frequencies (%) do not include missing data

The mean number of training hours was 942 (SD = 774). Table 2 presents information about the frequency of experience in personal therapy, current participation in supervision and possession of psychotherapy certification. More than half of the therapists (56.4 %) have no certificates given by general professional associations (e.g. Polish Psychological Association, Polish Psychiatric Association) or connected with a specific therapeutic orientation (e.g. Certificate in Group Analysis). However, most of the therapists who are not certified plan to obtain a psychotherapeutic certificate in the future (86.2 %).

We asked the respondents whether they identified with one orientation or with the eclectic/integrative approach. A total of 54 % of respondents endorsed the integrative or eclectic orientation, while the rest (46 %) reported commitment to only one theory. We also posed questions about the psychotherapeutic orientations they used (on average the therapists chose 2.8 single schools of psychotherapy) and one that was the most important to them. Table 3 presents the frequencies of the answers for the single schools of psychotherapy and the answers grouped into 4 main categories of psychotherapy.

**Table 3:** Schools of psychotherapy

Schools of psychotherapy	Used^a^		One that is the most important	
	N	%	N	%
Single schools of psychotherapy				
Psychodynamic	735	61.5 %	430	36.0
Cognitive-behavioral	482	40.3 %	126	10.5
Systemic	409	34.2 %	103	8.6
GESTALT	268	22.4 %	92	7.7
Existential	254	21.2 %	12	1.0
Psychoanalytic	247	20.7 %	151	12.6
Cognitive	201	16.8 %	5	0.4
Rogerian	187	15.6 %	34	2.8
Ericksonian	155	13.0 %	31	2.6
Behavioral	119	9.9 %	2	0.2
Process-oriented psychotherapy	100	8.4 %	32	2.7
Neurolinguistic programming	37	3.1 %	4	0.3
Other orientations	141	11.8 %	155	13.0
Uncommitted	11	0.9 %	19	1.6
Most important orientation grouped into categories:				
Psychoanalytic/psychodynamic	982	82.1 %	581	48.6
Cognitive-behavioral	802	67.1 %	133	11.1
Humanistic-existential	709	59.3 %	138	11.5
Systemic	409	34.2 %	103	8.6
Other approaches	444	37.1 %	241	20.2

^a^More than one answer was allowed, that is why the sum does not total 100 %

### Professional Environment and Patients Treated

Table 4 presents information about the form of treatment, individual or team work and the workplace where the psychotherapy is conducted. Interestingly, psychotherapy is offered, on average, in 2.1 places by one therapist. Moreover, Table 4 provides information about the therapist’s weekly workload (with the modal answer of 11–15 h per week) and any additional professional activities conducted by the therapists. The therapists were engaged, on average, in 2.5 professional activities that were other than psychotherapy.

**Table 4:** Characteristics of professional activity

Professional activity	N	%
Form of treatment^a^		
Individual psychotherapy	1179	98.6
Group therapy	420	35.1
Couple therapy	401	33.5
Family therapy	243	20.3
Individual/team work^a^		
Working individually	883	73.8
Working on a team	653	54.6
Workplace where the psychotherapy is conducted^a^		
Private psychotherapy center/Private practice	1028	86.0
Public counseling center	431	36.0
Psychiatric hospital	331	27.7
Non-governmental organization (NGO)	275	23.0
Educational counseling center	171	14.3
Public psychotherapy center	150	12.5
Non-psychiatric hospital	96	8.0
School or university	92	7.7
Weekly workload in hours		
1–5 h	180	15.1
6–10 h	264	22.1
11–15 h	271	22.7
16–20 h	206	17.2
21–25 h	138	11.5
26 h and more	137	11.5
Additional professional activities^a^		
Only psychotherapy	136	11.4
Conducting workshops	678	56.7
Counseling/Consultation	565	47.2
Providing diagnosis and assessment	429	35.9
Teaching	385	32.2
Leading support groups	227	19.0
Providing supervision	162	13.5
Scientific research	146	12.2
Mental health promotion	110	9.2
Administration	74	6.2
Human resources	65	5.4
Psychiatric counseling	57	4.8
Career counseling	56	4.7
Nursing	5	0.4

^a^More than one answer was allowed, that is why the sum does not total 100 %

A great majority of the therapists treated adult patients with anxiety or personality disorders. Table 5 presents, in detail, the groups of patients treated by the therapists. The group titled “other problems” includes the many answers that included, among others, clients with no psychiatric diagnosis but who were looking for help due to certain “life problems” they were dealing with.

**Table 5:** Characteristics of the treated patients

Characteristics of the treated patients	N	%
Age^a^		
Adults	1175	98.2
Adolescents	608	50.8
Elderly	229	19.1
Children	218	18.2
Diagnoses^a^		
Anxiety disorders	1120	93.6
Personality disorders	974	81.4
Psychosomatic disorders	903	75.5
Mood disorders	888	74.2
Violence victims	653	54.6
Eating disorders	610	51.0
Somatic problems	399	33.4
Sexuality problems	292	24.4
Other psychotic disorders	287	24.0
Schizophrenia	285	23.8
Addictions	269	22.5
Violence offenders	209	17.5
Organic disorders	156	13.0
Mentally handicapped	89	7.4
Other problems	264	22.1

^a^More than one answer was allowed, that is why the sum does not total 100 %

## Discussion

Our aim was to provide a contemporary portrait of persons conducting psychotherapy in Poland. A large and diversified sample of therapists working in diverse clinical settings was surveyed.

The results show that psychotherapy in Poland is performed mostly by women, as men constitute only 20 %. The proportion of gender varies among countries; some are dominated by women (e.g. the UK, Canada, Australia, Argentina, China, India; [11–16]; some by men (New Zealand, South Korea; [14, 17]; and in some the proportions are relatively equal (Spain, Germany, Switzerland, Norway; [18–20]. However, even in countries in which the profession is dominated by women, the proportions are smaller than in Poland. It seems that in Poland particularly women are more attracted to this profession than in other countries.

The average age of Polish therapists is 40. The results from other studies show that there are countries with younger populations of therapists (e.g. South Korea – 34, China – 37, Argentina – 37 [17]; [12, 16]), but in most of the surveyed countries the age of the therapists is higher (Spain, Germany, Switzerland, Norway, India, New Zealand, [14, 15, 18–20]; in some exceeding even 50 years (USA, Canada, UK, Australia; [11, 13, 14, 21]. The average age above 50 may reflect the fact that in those countries most people enter the profession later in life as a second career, which probably occurs rarely in Poland; for example, in a survey conducted among psychotherapists in the UK, 95 % of respondents had another profession prior to psychotherapy [11].

### Education, Training and Experience of the Therapists

The results of the survey show that almost all of the participants had an MA and 7.7 % held a PhD. All of the therapeutic associations in Poland (as is the case in most countries in Europe) require an MA degree; none of the countries requires a PhD.

In terms of educational background, Polish psychotherapy is dominated by psychologists – they constitute about two thirds of the sample. Psychotherapy seems to be a rather unpopular practice among psychiatrists because only about 5 % of respondents have a medical education. This result is in contradiction with the findings of Czabała and Brykczyńska [10] which were made more than a decade ago, as they found that in the field of Polish psychotherapy there is an over-representation of psychiatrists. The result that psychiatrists comprise a small fraction of Polish therapists is striking in light of the information that the Polish Psychiatric Association certified the greatest number of psychotherapists (mostly psychologists). In most countries, psychotherapy as a profession is dominated by psychologists (e.g. Spain, Switzerland, Norway, Canada, New Zealand, Argentina, India; [12, 14, 15, 18–20] However, in some countries the main group consists of psychiatrists (e.g. Germany, South Korea; [17–19]) or social workers (Australia; [13]). There are inconsistencies in the literature regarding the primary education of psychotherapists in USA. Orlinsky & Rønnestad [19] showed that the main group of therapists in the USA are psychologists, whereas Cook et al. [22] showed that these were mainly social workers. These differences across the world are connected with different traditions and qualification requirements for practicing psychotherapy. In many countries, a degree in psychology or medicine is a standard. Surprisingly, in our study the percentage of practitioners with a background in pedagogy is relatively high, and higher than of those with a medical background.

The results regarding training are optimistic. The mean number of training hours is above 900. It must be stated that this calculation has limited reliability, as for many participants it was probably difficult to estimate the exact number from memory, especially as many respondents had had their training many years ago.

The average experience of the therapists is about 10 years. The largest group (38.8 %) are established therapists with 7 to 15 years of experience. This variable is strongly connected with the mean age of the therapists. Other studies show that the average experience varies from 5.6 to 24.4 years in different countries [13, 14, 16, 17, 19, 20, 22]. The average experience of Polish therapists seems to be longer than that of therapists from South Korea, China and Germany, but shorter than that of therapists from Norway, Spain, Australia, New Zealand, USA and Canada. This can be seen as an argument for making the statement that the profession in Poland is quite well established.

Our data shows that the prevalence of the therapists’ personal therapy is high. Only one out of ten practitioners has not undergone any personal therapy. This result is optimistic and similar to other countries. There is little variation among other countries concerning this variable. Almost all (79–93 %) therapists in all of the surveyed countries underwent personal therapy [14, 18, 20, 23].

The results of our study show that the majority of respondents are engaged in clinical supervision. Only 5 % do not attend any supervision. The prevalence of supervision is similar to that in Germany and Switzerland, where 90 % of the therapists claim to have supervision [18], and is much higher than that in China, where only 64 % of the therapists have supervision [16].

We observed, however, that a relatively small percentage of the therapists (44 %) were certified. This means that many therapists are actively practicing therapy while being in training or having finished their training but have not decided to write their final thesis (usually a case study) and to take the final exam. It is difficult to compare this result with data from other countries due to the existence of many different forms of certification. One study showed that about 64 % of practitioners in Germany and about 85 % in Switzerland were certified [18]. Another study from China showed that in this country, 77 % of practitioners were certified [16]. The relatively low percentage of certified therapists may indicate a problem in the Polish therapeutic community. One explanation is that the external motivation to finish one’s training with a certificate is limited since it is not required by law, whereas it usually requires additional effort.

More than half of the therapists identify themselves with the integrative or eclectic orientation. This high percentage of eclectic or integrative therapists reflects the patterns that are observed in other countries. Integration and eclecticism is an emerging and continuing trend in psychotherapy [24]. In Germany and Switzerland [18], half of the therapists identify themselves with more than one orientation, while in the UK and USA even the majority do so [22, 25].

The psychotherapeutic domain in Poland is dominated by the psychodynamic-psychoanalytic theory and practice. More than 80 % of the therapists used psychodynamic/psychoanalytic concepts, and for almost half of them these were the most important in their work. Cognitive-behavioral (67 %) and humanistic-existential (59 %) concepts were also widely represented among the therapists, but these are used relatively infrequently (approximately 11 %) as primary schools of psychotherapy. Surveys conducted in other countries show that the most popular orientation in most countries is also psychodynamic; it is the primary orientation in Germany, Switzerland, Spain, Norway, Australia, South Korea, and Argentina (psychoanalysis) [12, 13, 18–20]. Cognitive-behavioral (cognitive and/or behavioral) is the most prevalent orientation in the USA ([19, 22], Canada, New Zealand, China and India [14–16]. The humanistic orientation is the primary orientation in the UK [25].

These results show that Polish therapists prefer classic approaches to psychotherapy. In light of the fact that there exists relative freedom (due to the lack of legal regulations), this result may be optimistic because it shows that experimental or dubious therapy methods are used infrequently.

### Professional Environment and Patients Treated

Almost all of the therapists conduct individual psychotherapy and about one third conduct group therapy and couples therapy; a less frequently used form of treatment is family therapy (20 %). The results of studies conducted in other countries show that individual therapy is the most popular therapy form in all of the countries studied (>90 % [15, 19, 20]. The other therapy forms show differences between countries, as in some countries other therapy formats (couple vs family vs group) are practiced considerably less often (e.g. South Korea: 17 % vs 20 % vs 37 %; Norway: 35 % vs 31 % vs 22 %), and in some countries almost as often as individual therapy (India: 91 % vs 90 % vs 75 %). This probably reflects the specialization level in the profession and places Polish therapists somewhere between those two extreme poles.

The results also show that half of the respondents work on a team. This is a good sign because of the supportive function of the team. Results from Germany and Switzerland show that therapists from those countries report working individually three times more often than on a team, at least when it comes to private practice [18]. The result reported in this study may also reflect the less individualistic character of Polish culture.

A private psychotherapy center/private practice was by far the most common practice site (86 %). The dominance of private practice is larger than in most other countries, e.g. South Korea, Germany, Norway, New Zealand, USA, Switzerland, or Australia [13, 14, 17–19, 22]. A similar rate to the one in this study was observed in Spain and Canada [14, 20]. It reflects the huge changes that occurred in Poland with the development of the free market. It is possible that freeing the market in connection with the emerging needs for therapy has initiated a developmental boom of the discipline.

At the same time, only 11.4 % of respondents described psychotherapy as their only professional activity, which is a rather small number. Half of the sample was additionally involved in conducting workshops and counseling/consulting. The proportion of practitioners working full time in the domain of psychotherapy as observed in other countries is much larger; for example, 84 % of psychologists practicing psychotherapy in the USA [26], 47 % of therapists in Australia [13] and 47 % in China [16] do so full time. Our question format did not allow them to, however, state whether those respondents who had claimed to be involved in other activities practiced these activities within or additionally to their full-time job. Comparing results from the US study [27], Polish therapists are less involved in doing research (12 % vs 30 %), diagnosis (35 % vs 60 %) and administration (6 % vs 39 %) than US therapists. In the US study, conducting workshops was not mentioned (it probably fit into the ‘other activities’ category), while in Poland it was the second most popular activity, thus indicating a characteristic feature of psychotherapy practice in Poland. The comparison is limited because the US study dealt only with therapists with a psychological background.

The modal workload of the therapists surveyed here (22.7 %) was between 11 and 15 h per week. This is much lower than in the USA (on average 22 h) or Canada (on average 20 h) but more than in New Zealand (12 h) [14]. It is difficult to compare this result with results from other countries because usually caseloads are measured instead of the number of sessions. This result is rather small but may be connected with another observation – that psychotherapy is the sole occupation for only a small percentage of our sample.

Almost all of the participants treat mostly adults, one half treat mostly adolescents and only one fifth treat mostly children and elderly people. Data from other countries show similar patterns [19]. These results may be connected with the demands of the market. Alternatively, they may reflect a deficiency in training possibilities. To our knowledge, there are only 3 full postgraduate training programs in Poland that are tailored to therapists who would like to work with children.

The most common clients that Polish therapists work with suffer from personality disorders, anxiety disorders, psychosomatic disorders and mood disorders. These results are comparable to those obtained in the study of Cook et al. [22]. In their study (based on additional data shared in personal communication), almost all of the surveyed therapists in the USA declare to work with mood/anxiety disorders (98.1 %). A total of 72.6 % of the American therapists worked with the clients with personality disorders, while in the Polish sample this number of therapists was 81.4 %. The slightly higher prevalence of personality disorders in the private practice of Polish psychotherapists may be connected with the fact that in Poland there is a high frequency of psychodynamic-oriented psychotherapists, so patients with personality disorders constitute the most common target group for this group of therapists. Almost one fourth of Polish therapists work with people diagnosed with schizophrenia, and this result is comparable with that of many Western countries [19]; for a detailed description of Polish psychotherapists working with persons with psychosis, see [28].

### Summary

As compared with previous reports, it seems that psychotherapy in Poland has made a huge step in its development. In 2001, Czabała and Brykczyńska [10] reported that psychotherapists in Poland had a much shorter training period and less supervision as compared to psychotherapists in Western countries. The results of our study, conducted a decade later, contradict these earlier observations. We found that persons who practice psychotherapy in Poland claim to have enough expertise and to be sufficiently trained. The reason for this may be that the majority of Polish psychotherapeutic associations has developed training programs that are more or less compatible with the requirements of the respective international associations regarding the certification of psychotherapists and supervisors. Our results also contradict opinions that have been raised in the general public in Poland that Polish psychotherapists are not professional (e.g. [29]).

To sum up, the results show that psychotherapeutic practice is well established in Poland. The way psychotherapy is practiced in Poland resembles that existing in Western countries. We found a common use of major therapy approaches and a high representation of eclecticism. The results show that Polish therapists prefer classic approaches, despite the relative freedom in choosing various approaches. It was also found that private practice was by far the most common type of practice. Polish therapists also seem to be well trained.

### Limitations

The study was not without limitations. First, it was based entirely on self-report data, thus it suffers from the problems of many other surveys. This problem may be particularly true for therapy orientations, especially among therapists who claim to work integratively or eclectically. It was not possible to recognize whether the orientation declared in the survey was connected with real expertise and competences. Adding objective measures based on observation of the therapeutic practice would be a valuable factor in future studies.

Another problem was that our sample was not random, so a generalization of the results on the whole population should be done with great caution. There is a lack of normative data on the actual population of therapists in Poland. Because of this we decided not to restrict the target population to selected professions (e.g. psychologists), psychotherapeutic associations or geographical regions. We tried to reach as many therapists as possible in diverse clinical settings.

Because we put much effort into receiving emails from Internet private practices, centers and various institutions, our sample may underrepresent practitioners who are not affiliated with any institution and who work without being visible on the Internet. We are aware that the results can also be biased due to the online form of responding to the survey, as there may be therapists for whom it is not easy to disclose information via the Internet. Another limitation was that the survey was time-consuming, which may have made it difficult to complete for some participants. Nevertheless, this study is the largest and most comprehensive study on psychotherapy practitioners in Poland to date.

## Footnotes

1 However, there are still some missing values in the analyzed database. Some respondents filled out the questionnaire in paper form and completed the whole survey but skipped certain questions. In the electronic form one could not go on to the next page of the questionnaire without having filled out all of the required questions on a given page.
